# Infectious Bursal Disease Virus Assembly Causes Endoplasmic Reticulum Stress and Lipid Droplet Accumulation

**DOI:** 10.3390/v15061295

**Published:** 2023-05-31

**Authors:** Yesica R. Frontini-López, Lautaro Rivera, Cristian A. Pocognoni, Julieta S. Roldán, María I. Colombo, Marina Uhart, Laura R. Delgui

**Affiliations:** 1 Instituto de Histología y Embriología de Mendoza (IHEM), Universidad Nacional de Cuyo, Consejo Nacional de Investigaciones Científicas y Técnicas (CONICET), Mendoza 5500, Argentina; 2 Facultad de Ciencias Médicas, Universidad Nacional de Cuyo, Mendoza 5500, Argentina; 3 Instituto de Virología e Innovaciones Tecnológicas, Centro de Investigaciones en Ciencias Veterinarias y Agronómicas, Instituto Nacional de Tecnología Agropecuaria (INTA), Consejo Nacional de Investigaciones Científicas y Técnicas (CONICET), Hurlingham 1686, Argentina; 4 Facultad de Ciencias Exactas y Naturales, Universidad Nacional de Cuyo, Mendoza 5500, Argentina

**Keywords:** infectious bursal disease virus, assembly, endoplasmic reticulum, lipid droplets

## Abstract

Gumboro illness is caused by the highly contagious immunosuppressive infectious bursal disease virus (IBDV), which affects the poultry industry globally. We have previously shown that IBDV hijacks the endocytic pathway to construct viral replication complexes on endosomes linked to the Golgi complex (GC). Then, analyzing crucial proteins involved in the secretory pathway, we showed the essential requirement of Rab1b, the Rab1b downstream effector Golgi-specific BFA resistance factor 1 (GBF1), and its substrate, the small GTPase ADP-ribosylation factor 1 (ARF1), for IBDV replication. In the current work, we focused on elucidating the IBDV assembly sites. We show that viral assembly occurs within single-membrane compartments closely associated with endoplasmic reticulum (ER) membranes, though we failed to elucidate the exact nature of the virus-wrapping membranes. Additionally, we show that IBDV infection promotes the stress of the ER, characterized by an accumulation of the chaperone binding protein (BiP) and lipid droplets (LDs) in the host cells. Overall, our results represent further original data showing the interplay between IBDV and the secretory pathway, making a substantial contribution to the field of birnaviruses–host cell interactions.

## 1. Introduction

IBDV, the etiological agent of Gumboro disease, is the best-characterized member of the *Birnaviridae* family, and the prototype of the genus Avibirnavirus [1]. Birnaviruses are unorthodox double-stranded RNA (dsRNA) viruses, which, due to their unique structural and replicative characteristics, have been proposed as an evolutionary connection between plus single-stranded RNA (+sRNA) and dsRNA viruses [2,3,4]. Three-dimensional reconstructions of birnavirus particles revealed that the viral capsids are formed by the protein VP2, while the genome, the RNA-dependent RNA polymerase (VP1), and the multifunctional protein VP3 are part of transcriptionally active filamentous structures called ribonucleoprotein complexes (RNPs) located inside the capsids [2,5]. The existence of RNPs in a dsRNA virus is a characteristic unique to the *Birnaviridae* family, which lacks the transcriptionally active core typically present in other dsRNA viruses [2,6,7].

The life cycle of IBDV begins with the attachment of the viral particles to the host cell. The precise receptor recognition is still not fully understood; however, several molecules such as HSP90, IgM, CD74, CD44, and α4β1 have been demonstrated to be involved in this viral step [8,9,10,11,12]. Whatever the molecule triggering the entry, the virus entry occurs by hijacking the macropinocytic pathway to end up within an early endosome characterized by the presence of the protein Rab5 [13]. There, the decreasing pH and calcium ions promote the viral capsid disassembly, releasing a small peptide, PEP46, with the ability to deform biological membranes [14]. To date, it is thought that this ability generates a continuity between the endosomal lumen and the cytoplasm, leading to the translocation of the viral RNPs to the cytoplasm by an as yet unknown mechanism. The next viral step was elucidated by analyzing the subcellular localization of the IBDV RNPs components, i.e., VP3, VP1, and dsRNA. It was shown that IBDV replication components are localized in the cytosolic side of endosomal compartments, creating cytoplasmic puncta, or inclusions, that appear shortly after infection and are termed “virus factories” (VFs) [15,16,17]. Recently, it has been shown that the VFs are molecular condensates with characteristics of liquid–liquid phase separation; they grow over the course of infection due to the synthesis of viral components and the coalescence of multiple VFs [18,19]. IBDV VFs have not been found to contain virus particles. Instead, transmission electron microscopy (TEM) reveals so-called paracrystaline virus arrays (PVAs), which are single-membrane structures wrapping up tightly packed, assembled virions in the cytoplasm of infected cells [20]. The mechanism by which the VPs lead to the formation of PVAs is completely unknown; however, due to partial evidence, in our laboratory, we support the hypothesis that the secretory pathway is involved in this viral process [16,21,22,23]. These PVAs are thought to be involved in virus spreading upon cell lysis; however, it has been demonstrated that, prior to lysis, viruses are exported out of the cell by a VP5-dependent mechanism and a network of single-membrane-containing viral particle-dependent mechanisms [20].

The secretory pathway is a network of compartments made up of the rough ER, the ER exit sites (ERES), the ER-GC intermediate compartment (ERGIC), the GC, and related transport vesicles [24]. This route is frequently exploited by viruses to accomplish several steps in their reproduction cycles [25,26,27,28,29,30,31,32]. Although some evidence has revealed a connection between components of the secretory pathway and the infectious cycle of *Birnaviridae* family members [16,21,22,23], this has not yet been investigated in depth.

In this study, we provide solid evidence, through a comprehensive examination, that the single-membranous compartments housing the newly assembled viral particles are in close apposition to the rough ER. Additionally, we present evidence of virus-induced ER stress, characterized by a significant accumulation of the chaperone BiP and LDs, later in infection. Altogether, our data further contribute to the knowledge of IBDV–intracellular trafficking interactions, demonstrating a novel interplay between IBDV and ER membranes within its infectious cycle.

## 2. Materials and Methods

### 2.1. Cell lines and Culture Conditions

Cell culture was conducted as previously described [13,15,16,17]. The cell lines Quail Muscle clone 7 myoblasts (QM7, ATCC CRL-1962) and human epithelial cervical cancer cells Henrietta Lacks (HeLa, ATCC CCL-2) were cultured in Dulbecco’s modified Eagle’s medium (DMEM; catalog number 12-800058; ThermoFisher Scientific, Buenos Aires, Argentina) containing 10% fetal bovine serum (Gibco; catalog number 15140122; ThermoFisher Scientific) under normal culture conditions at 37 °C and 5% CO_2_. In addition, QM7 cells were cultivated in the presence of 40 mM HEPES (catalog number 15630080, ThermoFisher Scientific) to offer additional buffering capacity.

### 2.2. Viral Stocks Production

Serotype I IBDV (Soroa strain) was propagated by employing QM7 cells as we have previously described [13,15,16,17]. Briefly, 70% of confluent QM7 cells were infected with IBDV at a multiplicity of infection (MOI) of 0.05 PFU/cell. At 72 to 96 h post-infection, the infected cells and supernatant were collected. The cells were subjected to three freezing and thawing cycles (−80 °C for 15 min/37 °C 5 min) and mixed with the supernatant for further clarification by centrifugation at 800× *g* at 4 °C for 15 min. The supernatant was combined with 20% polyethylene glycol 8000 and 3 M NaCl, followed by a 12 h incubation at 4 °C with gentle shaking. The viral particles were pelleted by centrifugation at 800× *g* for 30 min, and then the pellet was resuspended in PES buffer [25 mM piperazine-N, N=-bis (2-ethanesulfonic acid) (pH 6.2), 150 mM NaCl, and 20 mM CaCl_2_]. The viral suspensions were then aliquoted into cryovials and kept at −80 °C.

### 2.3. Antibodies

Mouse anti-βactin (1:2000 for Western blot, Sigma Aldrich, Buenos Aires, Argentina, catalog number A5441), rabbit anti-GFP (1:250 for immune electron microscopy, Abcam, catalog number ab290), mouse monoclonal anti-VP2 [(1:100 for indirect immunofluorescence (IIF)], whole rabbit antiserum immunized with recombinant VP1 and whole rabbit antiserum immunized with recombinant VP3 (1:1000 for Western blot and 1:500 for IIF) were gently gifted by Dr. José F. Rodriguez (CNB-CSIC, Madrid, Spain), alongside rabbit anti-BiP (1:300 for Western blot, Abcam, catalog number ab21685), rabbit polyclonal anti-ER antibodies (1:50 for IIF, a generous gift from Dr. Bruno Goud, Institut Curie, Paris, France), donkey anti-rabbit Alexa 488 (1:500 for IIF, ThermoFisher Scientific, catalog number A21206), goat anti-rabbit HRP (1:5000 for Western blot, Jackson Immunoresearch, catalog number A0545), goat anti-mouse HRP (1:10.000 for Western blot, Jackson Immunoresearch, catalog number A9044), goat anti-mouse Alexa 555 (1:500 for IIF, ThermoFisher Scientific, catalog number A32727) and goat anti-rabbit Gold 12 nm (1:40, Jackson Immunoresearch, catalog number 1111-205-144).

### 2.4. Pharmacological Inhibitors

Tunicamycin A treatment (Millipore-Sigma, Buenos Aires, Argentina, catalog number T7765), was used as previously described [33]. Briefly, 5 mg/mL Tunicamycin A was added to the media during the last 5 h of the experiment, in both mock- and IBDV-infected cells.

### 2.5. Oleic Acid Treatment and Viability Assay

QM7 cells were treated with oleic acid (OA, 0, 100, and 250 μM in DMSO) for 2 h to induce LDs accumulation, which was evaluated using Oil Red O staining, as described below, and previously [34]. To assess the potential impact of OA cytotoxicity in QM7 cells, an MTT [3-(4,5-dimethylthiazol-2-yl)-2,5-diphenyltetrazolium bromide] assay was performed following the method described by Kumar and collaborators [35], as we reported previously [23]. Briefly, QM7 cells were seeded in a 96-well plate to reach 80% confluency overnight (ON) in standard culture conditions. Then, the cells were incubated for 2 h either with 0, 100 or 250 μM OA. Since the OA stock was prepared in DMSO, the equivalent amount of this solvent was added to untreated cells for 2 h. After the specified incubation periods, the culture medium was removed and replaced with 100 μL of fresh Phenol red-free DMEM and 10 μL of 5 mg/mL MTT stock solution (prepared in PBS). Included was a negative control consisting of 10 μL of MTT stock solution added to 100 μL of media without cells. The plate was incubated at 37 °C for 4 h. The generated formazan was solubilized with DMSO as follows. In the wells, all but 25 μL of the medium were removed. With a pipette, 50 μL of DMSO was added to each well and thoroughly mixed. The plates were incubated at 37 °C for 10 min. Each sample was homogenized, and the absorbance was measured at 540 and 680 nm. Adjusted for non-specific background absorbance, the average value of Abs540 nm from MTT-only wells (without cells) was subtracted from all other Abs 540 nm values, which were divided by Abs 655 nm values.

### 2.6. Oil Red O Staining

LDs in mock and IBDV-infected QM7 cells were colored with Oil Red O (Biopack, Argentina) as detailed by Gojanovich and collaborators [34]. The cells were grown on coverslips in 24-well plates, rinsed three times with PBS containing 1 mM CaCl_2_ and 1 mM MgCl2, and then fixed with paraformaldehyde solution 4% (PFA) for 20 min. Oil Red O (0.35 % *w*/*v*) in isopropanol was diluted in MilliQ quality water (MQ, 6/4 ratio), filtered through a 0.2 μm nitrocellulose membrane, added to the fixed cells for 2 h at room temperature (RT) protected from light with gentle shaking, and subsequently rinsed exhaustively with MQ water. The samples were mounted with Mowiol 4-88 (Sigma-Aldrich, Buenos Aires, Argentina), as recommended by the manufacturer, for microscopy analysis. Finally, cell monolayers were analyzed using fluorescence microscopy. The LDs number per cell and size were assessed using Fiji-ImageJ2 open-source software [36]. Calibrated images were adjusted to facilitate the segmentation of LDs, which were aggregated in clusters, and the “analyze particles” function was applied to determine the number of LDs per cell, and size (setting from 0.1 μm to infinity). The area under the curve was calculated from the size frequency histogram of LDs (bin width = 0.5 µm) using GraphPad Prism v9 software, and was represented in the bar graph as the LDs area.

### 2.7. Plasmids and Transfection Methods

A plasmid encoding GFP-Sec61β was kindly provided by Alex Palazzo (University of Toronto, Toronto, Canada). The plasmid encoding GFP-KDEL was kindly provided by Dr Sergio Grinstein (University of Toronto, Toronto, ON, Canada). The plasmid encoding EGFP-Tip47 was kindly gifted by Dr Stefan Höning (Institute for Biochemistry I and Cologne Center for Molecular Medicine, University of Cologne, Cologne, Germany).

For transient transfections, QM7 cells were cultured to reach 80% confluence and transfected using Lipofectamine^TM^ 3000 (ThermoFisher Scientific, catalog number L300015) or FuGene HD (Promega, catalog number E2311), following the manufacturer’s recommendations. At 12 h p.t., the cells were fixed using 4% PFA for 15 min at RT, or infected with IBDV following specific experimental time-points.

### 2.8. Indirect Immunofluorescence

In a 24-well plate, QM7 or HeLa cells were cultured on 12 mm diameter coverslips. At the appropriate times p.i. or p.t., the monolayers were rinsed twice with PBS and fixed for 15 min at RT with 4% PFA solution. The monolayers were then permeabilized for 20 min at RT with 0.05% *w*/*v* saponin in PBS containing 0.2% *w*/*v* bovine serum albumin (BSA). For Oil Red O-stained sample immunofluorescence, we followed the procedure reported by Gojanovich and colleagues [34]. After Oil Red O staining, coverslips were permeabilized with 0.05% *w*/*v* saponin in PBS containing 0.2% *w*/*v* BSA for 20 min at RT and then treated as stated. The monolayers were then treated for 1 h 30 min at RT or ON at 4 °C with primary antibodies. After extensive washing with PBS, the monolayers were incubated for 1 h 30 min at RT with secondary antibodies. After several PBS washes, the cells were mounted with Mowiol, Mowiol with Hoechst, or Dako fluorescent mounting media, including DAPI. Finally, laser scanning confocal microscopy (LSCM) was utilized to examine the monolayers. Confocal microscope Olympus FluoView TM FV1000 (Olympus, Argentina) software FV10-ASW (version 01.07.00.16) or confocal microscope Nikon C1 (Nikon, Japan) software EZ-C1 was utilized. Using Adobe Photoshop CS5 (Adobe Systems, San Jose, CA, USA), data processing and analysis were conducted. The images for ER proteins were deconvoluted. To do this, we employed two specific plugins in ImageJ2: first, diffraction PSF 3D, and then parallel iterative deconvolution. Colocalization analysis between IBDV VP2 and ER signals was carried out by calculating the Pearson’s correlation coefficient using JACoP tool in FIJI-ImageJ2 open-source software. Utilizing combined 2 μm wide z-stacks, 3D reconstructions were carried out.

### 2.9. Western Blot

Using Laemmli sample buffer (0.5 M Tris pH 6.8, glycerol, 10% SDS, 2 mM DTT, and 5% *w*/*v* bromophenol blue), whole cell lysates were produced, and the proteins were denatured with heating at 95 °C for 10 min. Using 12% acrylamide gels for electrophoresis, proteins were separated and then transferred to Hybond-ECL nitrocellulose membranes (GE Healthcare, Chicago, IL, USA). The membranes were rinsed in PBS, blocked with 5% *w*/*v* non-fat milk in PBS for 2 h at RT or ON at 4 °C, and then incubated with the aforementioned primary antibodies ON at 4 °C. After three 15 min washes with 0.05% *v*/*v* Tween 20 solution in PBS, the membranes were incubated for 1 h 30 min at RT with the respective HRP-conjugated secondary antibodies. After extensive washes with 0.05% *v*/*v* Tween 20 solution in PBS, two chemiluminescent detection kits (WBKLS0100, Millipore or WBLUR0500, Merk-Millipore, Buenos Aires, Argentina) were used to identify immunoreactive bands, and data were acquired using an ImageQuant^TM^ LAS-4000 (Fujifilm, Tokyo, Japan). Using Adobe Photoshop CS5, the immunoreactive bands were analyzed and quantified.

### 2.10. Viral Titration by Plaque Assay

QM7 cells were grown in a 24 multi-well plate until 70% confluence. Then, they were infected with serial dilutions of IBDV in triplicate. After 1 h of viral adsorption at 37 °C, the monolayers were incubated with a 1:1 mix of DMEM 2×, and 4% low-melting-point agarose solution for 5 days at 37 °C. Finally, the monolayers were fixed with 10% formaldehyde solution for 2 h at RT and stained with 1% *w*/*v* crystal violet solution to identify lysis plaques for the estimation of viral titers (PFU/mL). For extracellular IBDV titration, the supernatants of infected cells were collected and used for the plaque assay as described [16].

### 2.11. Transmission Electron Microscopy

QM7 cells were grown in 100 mm culture dishes to 70% confluence, and then infected with IBDV at an MOI of 1 PFU/cell. At 48 h p.i. the cells were fixed with 2.5% glutaraldehyde in sodium cacodylate buffer, 0.15 M pH 7.4, for 1 h at RT. Subsequently, the cells were pre-infiltrated in a 2% low melting point agarose solution and post-fixed with 2% osmium tetroxide (OsO4) for 2 h at RT. Following dehydration using incubation with increasing concentrations of acetone solutions (30, 50, 70, 90 and 100%), the samples were embedded in low viscosity Spurr’s epoxy resin for 24 h at 56 °C. Finally, ultrathin sections between 60–70 nm were made using an ultramicrotome (Leica Ultracut R) and stained with lead citrate (0.5% *w*/*v*) and uranyl citrate (2% *w*/*v*) solutions. Images were acquired employing an electron microscope (Zeiss EM 900) integrated with a high-resolution camera (CCD Gatan SC1000) and processed with Adobe Photoshop CS5 (Adobe Systems).

### 2.12. Cryo-Immunoelectron Microscopy

QM7 cells were grown in 100 mm culture dishes until 70% confluence, and then transfected with the construct pGFP-Sec61β using FUGENE HD, following the manufacturer’s instructions. At 12 h p.t., the cells were infected with IBDV at an MOI of 1 PFU/cell. At 48 h p.i., the cells were fixed with 4% PFA, 0.2% *v*/*v* glutaraldehyde and 0.1% *w*/*v* sucrose in Sorensen’s buffer 0.2 M, pH 7.4 (0.2% NaH_2_PO_4_-H_2_O; 2.3%Na_2_HPO_4_ aqueous solution) ON at 4 °C. After incubating with 50 mM glycine solution in Sorensen’s buffer for 30 min, the cells were embedded in 10% *w*/*v* gelatin. Gelatin blocks were embedded in 2.3 M sucrose solution at 4 °C for 2 h and subsequently frozen in liquid nitrogen. Ultrathin cryosections were obtained by employing a cryo-ultramicrotome (Leica Ultracut R). The sections were recovered in 2% *w*/*v* methylcellulose; 2.3 M sucrose solution (*v*/*v*), and mounted on 300 mesh Formvar-covered grids (Ted Pella, Redding, CA, USA). For immunogold labeling, ultrathin sections were blocked by incubating them with 5% BSA solution in Sorensen’s buffer and then incubating with anti-GFP primary antibodies diluted to 1:250 in Sorensen’s buffer, or with Sorensen’s buffer alone (i.e., the secondary antibodies’ control condition). After extensive washes with Sorensen’s buffer, grids were incubated with 1:20 dilution of anti-rabbit secondary antibodies conjugated with 12 nm Gold particles. Finally, sections were stained with lead citrate (0.5%) and uranyl citrate (2%) aqueous solutions and added, on ice, to a 9:1 mixture of 2% methylcellulose and 3% uranyl acetate. Images were acquired employing an electron microscopy Hitachi H7500 and processed with Adobe Photoshop CS5 (Adobe Systems).

### 2.13. Statistical Analysis

Statistical analysis was carried out with a Student’s *t*-test using Ky-Plot software version 2.0 beta 15. A 95% confidence interval was set to determine statistical significance. All data shown are mean ± standard deviation (SD) from three independent experiments.

## 3. Results

### 3.1. Newly Assembled IBDV Particles Are Enclosed within Single-Membrane Compartments Closely Associated with ER Membranes

**Figure 1:**
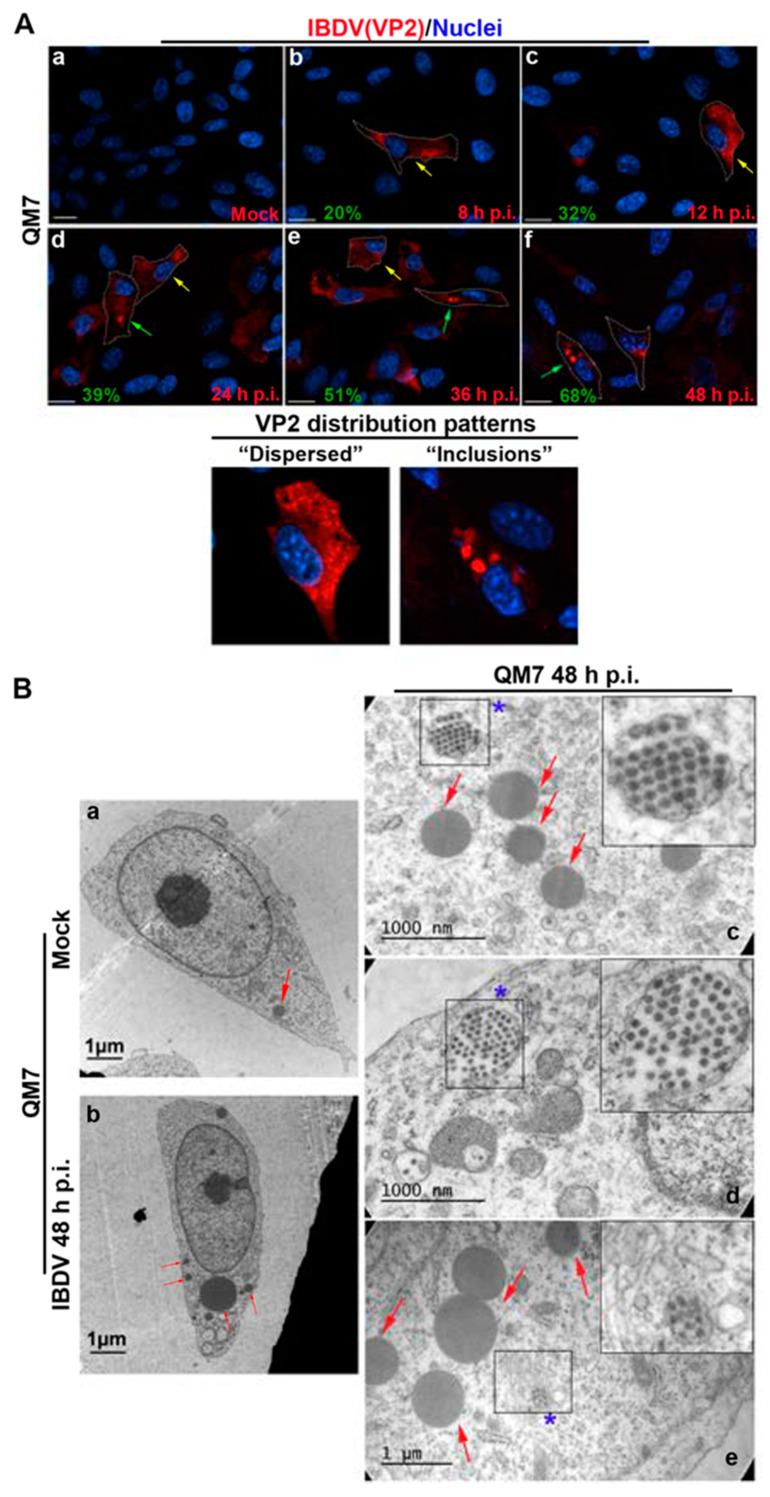
Subcellular localization of IBDV viral progeny. (**A**) VP2 subcellular distribution in IBDV infection. QM7 cells were infected with IBDV at an MOI of 1 PFU/cell or maintained in mock condition. At 8, 12, 24, 36, and 48 h p.i., the monolayers were processed using IIF to determine the subcellular distribution of the VP2 capsid protein and analyzed using SDCM. Anti-VP2 and secondary antibodies were used as described in Section 2. (**Aa**–**Af**) are representatives of three independent trials. Yellow arrows on (**Ab**–**Ae**) indicate cells containing newly synthetized VP2 proteins detected from 8 h p.i., mainly dispersed in the cytoplasm, and green arrows on (**Ad**–**Af**) indicate cells showing VP2 localized in large cytoplasmic inclusions. Written in green, at the bottom-left side of each image, we show the percentage of cells with VP2 forming inclusions. Below the main panel, two zoom-in views of the characteristic “dispersed” or “inclusions” distribution patterns from VP2 are shown. Scale bars represent 10 μm. (**B**) Ultrastructural analysis of IBDV infected cells. QM7 cells were infected with IBDV at an MOI of 1 PFU/cell or maintained in mock condition. At 48 h p.i., the monolayers were processed using TEM, as described in Section 2. Images are representative of three independent trials. The left panel shows a complete mock-infected (**Ba**) or IBDV-infected (**Bb**) cell at low magnification. The right panel shows three images (**Bc**–**Be**) corresponding to higher magnification images from IBDV-infected cells. Red arrows show LDs. Blue stars show newly synthesized viral particles organized within single-membrane compartments.

**Figure 2:**
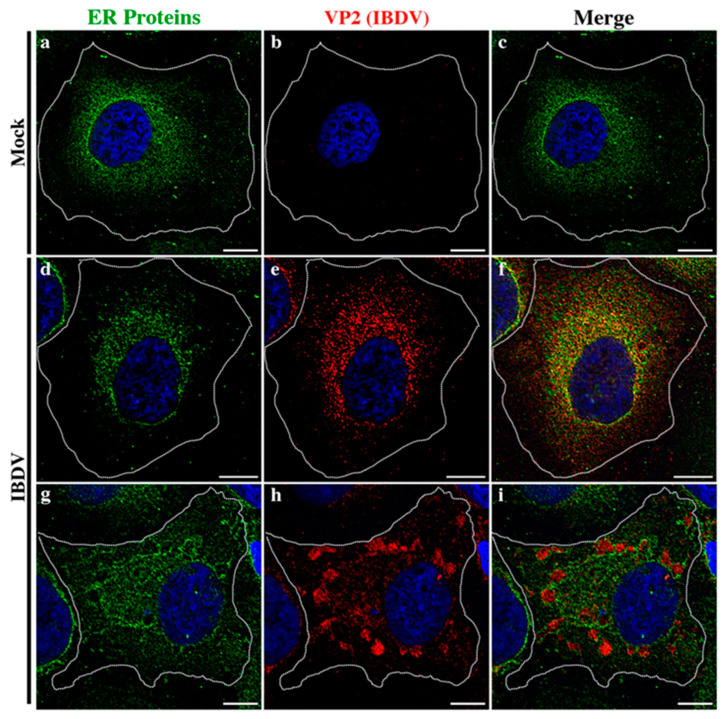
Relationship of IBDV viral progeny with the host ER. Subcellular distribution analysis of IBDV VP2 employing anti-ER antibodies. HeLa cells were infected with IBDV at an MOI of 1 PFU/cell or maintained in control media. At 36 h p.i., the monolayers were processed using IIF to determine the subcellular distribution of IBDV VP2 and ER proteins. ER proteins were detected by employing a polyclonal anti-ER serum, as described in Section 2. Finally, the monolayers were analyzed by SDCM. Panels (**a**–**c**) show the distribution of ER proteins in mock-infected condition. Panels (**d**–**f**) show the colocalizing dispersed distribution of both ER and VP2. Panels (**g**–**i**) show the VP2 large inclusions surrounded by ER-derived membranes. Bar scales represent 10 µm.

**Figure 3:**
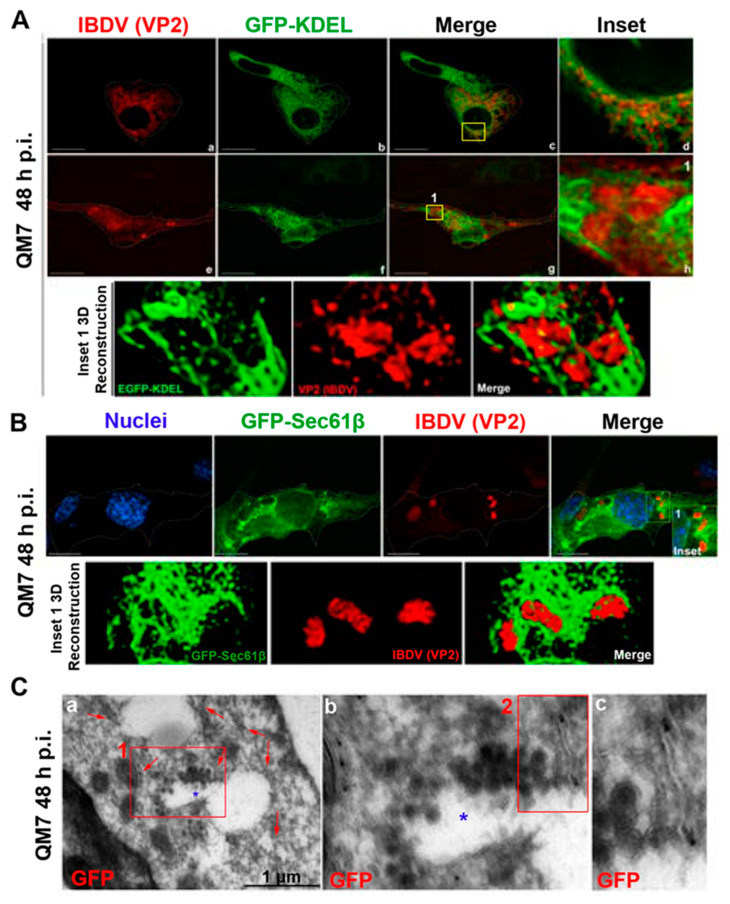
Relationship of IBDV viral progeny with the host ER. (**A**,**B**) ER re-distribution in IBDV infected cells. QM7 cells were transfected with (**A**) pGFP-KDEL (ER lumen marker) or (**B**) pGFP-Sec61β (ER membrane marker) and 12 h p.t. the cells were infected with IBDV at an MOI of 1 PFU/cell. 48 h p.i. the monolayers were processed by IIF to detect VP2 as described in Section 2, and analyzed by SDCM. Images cor-respond to merges of Z stacks representatives of three in-dependent trials. Images (**a**–**d**) show small VP2 aggregates dispersed in the cytoplasm without association with the GFP-KDEL-derived signal, and images (**e**–**h**) show VP2 lo-calized in large cytoplasmic inclusions surrounded by the GFP-KDEL-derived signal. The yellow square numbered 1 on image g is represented enlarged on image h and its 3D re-construction below. The yellow square numbered 1 on B represents the area within the 3D reconstruction shown below. 3D reconstructions of inset 1 were obtained as de-scribed in Section 2. Scale bars represent 10 μm. (**C**) Spatial relationship between virus-containing compartments and the ER membranes. QM7 cells were transfected with pGFP-Sec61β and 12 h p.t., infected with IBDV at an MOI of 1 PFU/cell. 48 h p.i. the monolayers were processed by cryo-immunoelectron microscopy to detect ER membranes using anti-GFP antibodies as described in Section 2. The red square numbered 1 on image (**a**) is represented enlarged in image (**b**). The red square numbered 2 on image (**b**) is repre-sented enlarged in image (**c**). Red arrows show ER mem-branes with gold particles. The blue star shows a virus-es-containing compartment.

In the context of a recent analysis of IBDV exit, Méndez and collaborators [20] performed TEM analysis of IBDV-infected cells and observed single-membrane structures wrapping up PVAs in the cytoplasm of infected avian cells. However, the subcellular origin and the membrane nature of those structures are still unknown aspects of IBDV assembly and cellular egress. The cellular secretory pathway, which transfers proteins and membranes from the ER through the GC to the plasma membrane, represents a perfect platform for new virion assembly, maturation and release. To address this possibility, we initially focused on VP2, the only detectable component of the newly assembled viral particles. First, to determine the optimal time-point of infection for subsequent studies, we evaluated the kinetics of VP2 distribution throughout 48 h of infection. We assumed that the PVAs mentioned above are represented by the cytoplasmic inclusions stained by VP2 antibodies in indirect immunofluorescence (IIF) images. QM7 cells were infected with IBDV at an MOI of 1 PFU/cell or maintained in control medium (mock-infected), and at different time-points (8, 12, 24, 36 and 48 h), were processed by IIF, using specific anti-VP2 monoclonal antibodies. As depicted in Figure 1A, the newly synthetized VP2 proteins were detected from 8 h p.i. mainly dispersed in the cytoplasm (indicated by yellow arrows on Figure 1A(b–e)). At 24 h p.i., a percentage of infected cells showed VP2 localized in large cytoplasmic inclusions, a pattern that became particularly abundant at 36 and 48 h p.i. (indicated by green arrows on Figure 1A(d–f)). Hence, based on these observations, we selected 48 h p.i. for further studies in order to assess the role of the ER in IBDV assembly. Next, to gain insight into the viral assembly in our system, we performed TEM analysis. QM7 cells were infected with IBDV at an MOI of 1 PFU/cell or maintained in control medium (mock-infected). At 48 h p.i., the monolayers were processed by TEM, as described in Section 2. We observed multiple viral particles tightly arranged in PVAs, as previously observed by other authors [20,37], enclosed by single-membrane compartments (Figure 1B(c–e)). An additional frequent observation made in this study was a close apposition of PVAs with rough ER, albeit without evident physical communications between them (blue stars in Figure 1B(c–e)). Additionally, and surprisingly, in IBDV-infected cells, we consistently found multiple LDs that were heterogeneous in size (red arrows on Figure 1B(b,c,e)), in comparison to mock-infected cells, where we only eventually observed organelles of smaller sizes (red arrows on Figure 1B(a)). Subsequently, we employed anti-ER proteins and VP2 antibodies on IIF assays to analyze the subcellular distribution of newly assembled viral particles in relation to the host ER. First, we employed a whole rabbit antiserum raised against an entire lysate of ER membrane-isolated proteins [38]. As depicted in Figure 2, we observed two well-differentiated distributions of VP2 regarding the ER: a colocalizing dispersed distribution of both ER and VP2 with a Pearson’s colocalization coefficient of 0.4487 +/− 0.13 (Figure 2d–f), or VP2 large inclusions surrounded by ER-derived membranes (Figure 2g–i), which we attribute to a later stage of infection, when PVAs had already been formed. Considering the results shown in Figure 1b and Figure 2, we hypothesized that the ER participates in the assembly of the newly generated viral progeny. Therefore, we used two plasmids to over-express fluorescent proteins which specifically localize to the rough ER compartments. The first one was pGFP-KDEL, developed as a marker of the ER lumen [39]. This construction encodes a fusion protein that contains the KDEL signal peptide. The KDEL sequence acts as a retention signal for proteins residents of the ER, preventing their secretion, and facilitating their re-capture if they are accidentally exported out of the ER [40,41]. To mark the ER membrane, we employed the construction, pGFP-Sec61β [42]. Sec61β is a transmembrane protein and a component of the translocon, a protein complex involved in the recognition and transport of neo-synthesized polypeptides from ER-associated ribosomes [41]. Thus, QM7 cells were transfected with the plasmids mentioned above, and at 12 h p.t. were infected with IBDV at an MOI of 1 PFU/cell. Afterwards at 48 h p.i., the monolayers were processed via IIF using specific anti-VP2 monoclonal antibodies. Figure 3A shows VP2 adopting two different patterns of distribution at 48 h p.i., one forming small aggregates dispersed in the cytoplasm without association with the GFP-KDEL-derived signal (less than 5% of the infected cells) (Figure 3A(a–d)), and another one wherein VP2 localized in large cytoplasmic inclusions surrounded by the GFP-KDEL-derived signal. In this second situation, a concomitant reorganization of the ER surrounding the large VP2 cytoplasmic inclusions seems to occur (Figure 3A(e–h)). We also performed 3D reconstruction from IIF images with higher resolution, and confirmed the notion that VP2 cytosolic inclusions are not located within the ER, but in close apposition to the ER membranes. Similar observations were made when we employed the ER membrane marker, GFP-Sec61β (Figure 3B). Altogether, our observations strongly suggest that VP2 cytoplasmic inclusions, which we assume to be the viral assembly sites, are not within or enclosed by rough ER membranes, but rather are in close apposition to them, promoting a strong re-distribution of these membranes around the viral assembly sites. Afterwards, we analyzed the spatial relationship between the single-membrane compartments containing PVAs and the ER membranes. QM7 cells were transfected with pGFP-Sec61β, and at 12 h p.t., the cells were infected with IBDV at an MOI of 1 PFU/cell. At 48 h p.i., we performed cryo-immunoelectron microscopy employing anti-GFP specific antibodies to detect rough ER membranes in transfected cells. Figure 3C shows a close apposition (average 0.21 +/− 0.01 µm in distance) between PVAs and rough ER membranes, as indicated by the red arrows on image a. These observations reinforced our previous results, and demonstrated that newly synthesized viral particles are assembled inside single-membrane compartments found in close apposition to rough ER.

### 3.2. IBDV Infection Induces LDs and Chaperone BiP Accumulation in Host Cells

**Figure 4:**
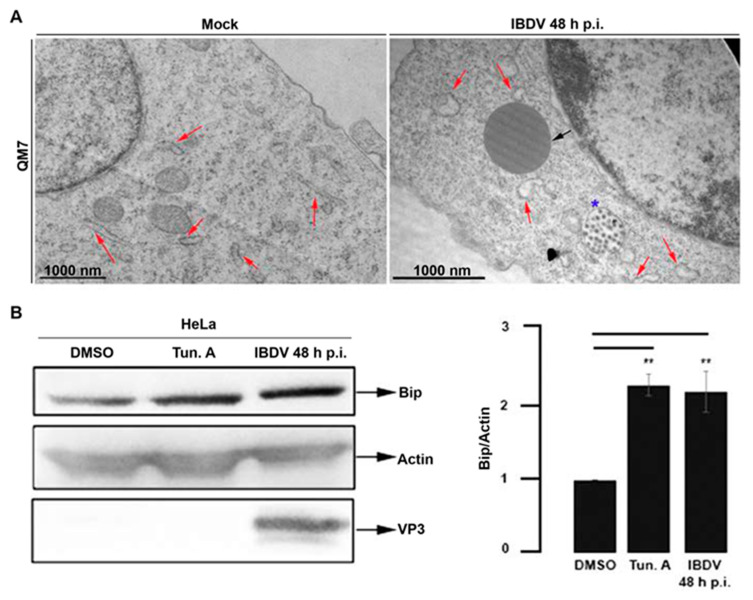
Analysis of ER stress in IBDV infected cells. (**A**) Ultrastructure of the ER in IBDV infected cells. QM7 cells were infected with IBDV at an MOI of 1 PFU/cell or maintained in mock condition. At 48 h p.i., the monolayers were processed using TEM, as described in Section 2. Images are representative of three independent trials. Red arrows show ER, and the black arrow shows a LD. The blue star shows a viral containing compartment. (**B**) BiP protein level in IBDV infected cells. HeLa cells were infected with IBDV at an MOI of 1 PFU/cell until 48 h p.i. Additionally, a set of non-infected HeLa cells were incubated with control media (DMSO) or 5μg/mL Tun. A for the last 5 h of the experiment. The monolayers were processed using Western blotting to determine the intracellular level of BiP protein, actin (as loading control) and VP3, as described in Section 2. The Western blot image corresponds to an experiment representing four independent trials. The data shown in the normalized bar graph correspond to four independent trials. Error bars show SD, ** *p* ≤ 0.05.

As we mentioned before, ultrastructural analysis of avian-infected cells revealed a large accumulation of LDs, some of them larger than 1 μm (Figure 1B). On the other hand, we observed a conspicuous re-distribution of ER membranes around the PVAs, characterized by a profuse modification of the typical ER reticular pattern (Figure 2 and Figure 3). We hypothesized that both observations might be a manifestation of virus-induced ER stress that may lead to LDs accumulation. The production of a large number of new virions within a host cell causes excessive stress on the protein folding machinery of the host ER [43]. In turn, the accumulation of LDs is often observed when ER stress is induced [44,45,46]. To further assess the ER re-organization, we performed TEM in mock- and IBDV-infected cells. A well-documented ultrastructural feature of ER stress is lumen dilation [47], a phenotype also described for viral infections which induce ER stress [48,49]. Thus, QM7 cells were infected with IBDV at an MOI of 1 PFU/cell or maintained in control media (mock-infected). At 48 h p.i., the cells were processed by TEM. Figure 4A clearly shows both a homeostatic ER morphology in mock-infected cells (Figure 4A, red arrows, left image) and dilatations of the ER, characterized by the enlargement of ER lumen [50,51,52,53], in IBDV-infected cells (Figure 4A, red arrows, right image), also suggesting IBDV-induced ER stress. The average width of ER cisternae in mock and IBDV-infected cells was determined and found to be 8 nm +/− 3.6 nm and 13 nm +/− 5.2 nm, respectively. On the host cell side, to survive to the ER stress, a complex and highly conserved cellular response called the unfolded protein response (UPR) is mounted [27]. One of the UPR features is the overexpression of ER chaperones. Thus, we determined the intracellular level of the chaperone protein BiP in mock- and IBDV-infected cells. BiP is an ER resident chaperone that plays a crucial role in the quality control of protein folding and the UPR response [54]. During UPR activation, an induction of the BiP expression takes place. Therefore, the intracellular level of BiP is an indicator of UPR activation. An increased level of BiP expression has been reported for many viruses that induce ER stress, such as respiratory syncytial virus [55], hepatitis C virus [56], bovine viral diarrhea virus [57] and Japanese encephalitis virus [58]. Thus, HeLa cells were infected with IBDV at an MOI of 1 PFU/cell, or maintained in control media (mock-infected, DMSO-treated). As a positive control for UPR activation, cells were treated with 5 μg/mL Tunicamycin A (Tun. A), which was added to the media during the last 5 h of the experiment in mock-infected cells. Tun. A is a widely used ER stressor which inhibits N-glycosylation and consequently induces the UPR response [59]. The monolayers were analyzed using Western blotting with anti-BiP and anti-VP3 specific antibodies. As expected, we observed a significant increase in the intracellular level of BiP after Tun. A treatment in comparison with DMSO-treated mock-infected cells (Figure 4B), suggesting the activation of the UPR response. Similarly, we observed a significant increase in the intracellular level of BiP protein in IBDV-infected cells, suggesting the virus-induced UPR activation as well (Figure 4B). Taken together, these results strongly point to the notion that IBDV infection triggers the ER stress at late times of infection, with consequent and characteristic UPR activation. 

**Figure 5:**
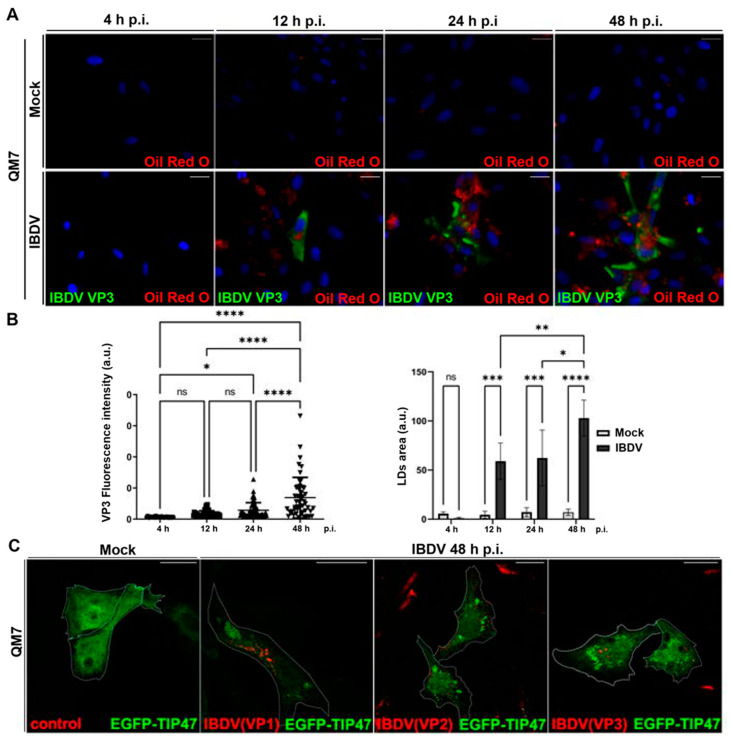
Accumulation of LDs in IBDV-infected cells. (**A**) Kinetic of LDs accumulation during IBDV infection. QM7 cells were infected with IBDV at an MOI of 1PFU/cell or maintained in mock condition. At 4, 12, 24, and 48 h p.i., the cells were stained with Oil Red O and subsequently subjected to the IIF technique to detect VP3, as described in Section 2, and analyzed using fluorescence microscopy. Nuclei were stained in blue using DAPI. Images are representative of three independent trials. Bar scales represent 10 μm. (**B**) Quantification of VP3 and LDs accumulation in IBDV infected cells. Using the images from the experiment represented by panel (**A**), both the VP3-stained infected cells and the LDs number and size were assessed using Fiji-ImageJ2 open-source software, as described in Section 2. The bar graph on the left corresponds to the quantitative analysis showing the kinetics of the infection rate (a.u.: VP3 fluorescence intensity/cell), and the right panel contains the bar graph showing LDs area (a.u.: arbitrary units). Error bars show SD,* *p* < 0.1, ** *p* < 0.01; *** *p* < 0.001 and **** *p* < 0.0001. (**C)** Subcellular distribution of IBDV viral proteins and LDs in IBDV infected cells. QM7 cells were transfected with pEGFP-TIP47, and at 12 h p.t., the cells were infected with IBDV at an MOI of 1 PFU/cell or maintained in mock condition. At 48 h p.i., the monolayers were stained with Oil Red O and subsequently subjected to the IIF technique to detect the structural viral proteins VP1, VP2, and VP3, as described in Section 2. Finally, the monolayers were analyzed using LSCM. Images are representative of three independent trials. Scale bars represent 10 μm.

In this context, we decided to investigate virally induced LDs accumulation in a more detailed manner. Thus, QM7 cells were infected with IBDV at an MOI of 1 PFU/cell, and at different timepoints (4, 12, 24, and 48 h), the cells were processed with IIF, using specific anti-VP3 monoclonal antibodies to detect infected cells. Additionally, before IIF, we employed Oil Red O to stain LDs on the same cells (Oil Red O–IIF double-staining), as we described earlier [34]. We observed a significant increase in the number of LDs in infected monolayers, from 12 h p.i., with a significant increase in LDs size and number (total area) towards 48 h p.i. (Figure 5A,B). Next, since many viral pathogens hijack LDs as physical platforms for their replication cycles [26,60,61], we aimed to analyze the subcellular distribution of structural viral proteins (VP1, VP2 and VP3) regarding LDs. For this objective, we employed the plasmid pEGFP-TIP47, which encodes a fusion protein widely used as LDs marker. TIP47 is a membrane protein associated with the LDs surface, and involved in their biogenesis [62,63]. Subsequently, QM7 cells were transfected with pEGFP-TIP47, and at 12 h p.t., the cells were infected with IBDV at an MOI of 1 PFU/cell. At 48 h p.i., the cells were processed with IIF, using anti-VP1, VP2 and VP3-specific antibodies. Figure 5C depicts that while EGFP-TIP47 showed a cytoplasmic distribution in mock-infected cells, it remained mainly associated with LDs in virus-infected cells. Additionally, when we analyzed the subcellular distribution of viral proteins in transfected cells, we did not observe co-localization between the viral components and LDs. These results suggest that while IBDV specifically induces the accumulation of LDs, its structural components do not interact with the lipidic organelles, strongly pointing to the accumulation of LDs as a secondary-to-ER stress response in IBDV infection. Taken together, our results suggest that IBDV promotes ER stress in the host cells, which in turn respond through BiP and LDs accumulation.

### 3.3. LDs Do Not Have a Role in IBDV Replication

**Figure 6:**
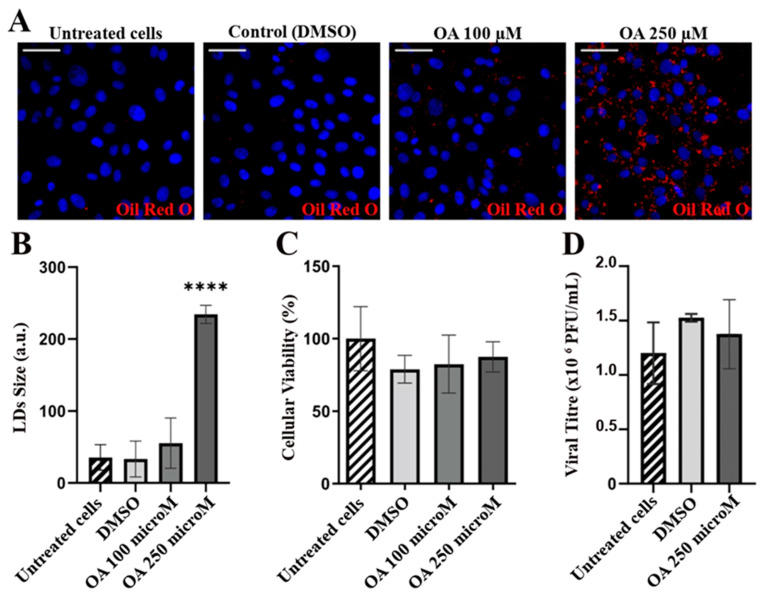
LDs accumulation does not have a role in viral infection. (**A**) Oleic Acid induction of LDs in QM7 cells. QM7 cells were treated with Oleic acid [OA, 0 (DMSO), 100, and 250 µM in DMSO] for 2 h to induce LDs accumulation, which was evaluated using Oil Red O staining (red) as described in Section 2, and analyzed using fluorescent microscopy. Nuclei were stained in blue using DAPI. Images are representative of three independent trials. Scale bars represent 12 μm. (**B**) LDs accumulation quantitation. The images from the experiment (**A**) were analyzed, and the LDs size and number were assessed using Fiji-ImageJ2 open-source software, as described in Section 2. The bar graph shows the LDs area (a.u.: arbitrary units) for each condition. Error bars show SD, **** *p* < 0.01. (**C**) MTT viability assay. QM7 cells were cultured and treated as in (**A**). After 2 h, the MTT assay was performed as described in Section 2. The data in the bar graph are representative of three independent MTT trials. Error bars show SD, no significant differences were observed. (**D**) IBDV replication and IBDV viral titration in untreated, DMSO-treated and OA 250 µM-treated QM7 cells. The data in the bar graph are representative of the three independent trials. Error bars show SD, and no significant differences were observed.

The interplay between the LDs and the viral infectious cycles has recently become a well-explored field, with the LDs serving not only as a scaffold for replication and assembly, but also as a source of energy or membrane lipids [64,65,66,67,68]. Having observed a significant increase in the number of LDs in IBDV-infected cells and discarded their role as assembly platforms, we sought to analyze their potentially beneficial role in the viral cycle. To this end, we designed an assay with an opposite rationale wherein we first induced the accumulation of LDs and then infected the already overloaded cells. If the accumulation of LDs had a role in the viral cycle, a higher infective viral progeny would have been expected. So, we first verified that QM7 cells were able to accumulate LDs after treatment with the inducer OA. To this end, 0 (DMSO), 100 and 250 μM OA over 2 h were assessed for both LDs accumulation induction (assessed by Oil Red O staining) and cytotoxicity using the MTT method. We observed that 250 μM OA produced a significant accumulation of LDs (Figure 6A,B). Additionally, neither treatment produced a significant loss in cellular viability (Figure 6C). Then, the cells were infected with IBDV at an MOI of 1 PFU/cell, and at 48 h p.i., the supernatants were recovered and the infective virions titrated by plaque assay. As shown in Figure 6D, non-significant differences were observed in the infective viral progenies, reinforcing the notion that LDs accumulation in IBDV occurs as a consequence of ER stress, instead of having a role in the viral cycle.

## 4. Discussion

RNA viruses multiply in association with host cell endomembranes, which they alter to act as hiding niches, thereby evading the antiviral machinery of the host cell [32,69]. These structures, known as VFs, are scaffolds for genome replication and viral morphogenesis [70,71], wherein the viral replication machinery is inserted into single- (*Flaviviruses*, *Togaviridae*, *Bromoviridae*, *Nodaviridae*) or double-membrane vesicles (DMVs) (*Nidovirales*, *Picornaviridae* and *Hepaciviruses*) that may associate with different organelles such as the ER, mitochondria, endo-lysosomal compartment, GC, etc. The single-membrane vesicles consist of spherules produced by the invagination of specific host organelles, such as the ER, mitochondria, or endolysosomes, which remain connected to the cytosol by a narrow channel, thereby facilitating the import of metabolites and export of newly synthesized positive-sense RNAs to the cytosol for translation and packaging of new virions. In contrast, the DMVs are formed by complex clusters of vesiculotubular membranes with various structural components [72,73,74,75,76]. All DMV-inducing viruses studied to date appear to target membranes of the secretory pathway. In the past decade, advancements in electron microscopy (EM) imaging and EM sample preparation have substantially increased our knowledge of the VFs of DMV-inducing viruses. Specifically, electron tomography has provided extraordinarily detailed 3D images of these VFs, and revealed their unique structures. DMV biogenesis is a mechanistically complex process that involves multiple membrane-remodeling phases. Depending on the DMV topology, these include the induction of positive and negative curvature, membrane paring, membrane fission events and/or a combination of several of these with intermediate structures. For example, for several picornaviruses, it has been possible to clearly identify intermediates in DMV formation, supporting a model in which single membrane structures transform into membrane-paired cisternae that then curve and bind to form DMVs [77,78,79]. Early formation of profuse single-membrane structures is also characteristic of hepatitis C virus and norovirus infections, and DMV biogenesis pathways similar to those of picornaviruses have been proposed [80,81,82]. Diverse lines of evidence point to an alternate DMV biogenesis pathway for nidoviruses, in which segments of paired ER membranes bend progressively to form a DMV. Putative intermediates in this transformation have been observed in arterivirus-infected cells and, for both arteriviruses and coronaviruses, under the conditions in which this transformation is induced [83].

In order to decipher the membranous compartment assisting IBDV for assembly and host cell exit, we focused on the ER, mainly for two essential reasons: (i) the ER is the most often usurped intracellular organelle targeted by viruses for productive replication by virtue of its great membranous source; and (ii) observations from our laboratory strongly point to the ER-derived membranes as those involved in generating viral cytoplasmic assembly (VP2-stained inclusions) and further release compartments (Figure 2). For those experiments, we employed a whole rabbit antiserum raised against an entire lysate of ER membrane-isolated proteins [38], and observed a precise distribution of both ER- and VP2-derived signals, wherein viral inclusions where tightly associated with (Figure 2d–f) or surrounded by ER-derived fluorescent stain (Figure 2g–i). This was the reason that we also chose to demonstrate the ER membrane of origin enclosing the viral assembly compartments using more specific rough ER markers such as pGFP-Sec61β and GFP-KDEL probes. However, our studies indicated that the viral cytoplasmic inclusions were not within or enclosed by rough ER membranes, but rather in close apposition to them, promoting a strong re-distribution of ER membranes around the viral assembly sites as well (Figure 2). Taking these observations together with our recently demonstrated requirement of the Rab1b- GBF-1-ARF1 secretory pathway axis for the replication cycle [23], we hypothesize that intermediate ER-GC compartments, (ERGIC) or vesicular tubular clusters (VTCs), are likely to be the sites wherein the virus hijacks the membrane to give rise to the single-membranous compartment surrounding the viral assembly sites. Indeed, KDEL-containing proteins cycle through intermediate compartments between the GC and ER [84]; therefore, some of the staining we observed may be due to GFP-KDEL in those compartments. However, more specific probes will be required for an exact definition of the membrane source.

In a recent publication, the group of Dr. Xiaomei Wang reported that IBDV infection promotes autophagy during the late stage of infection, which in turn facilitates viral maturation and release [85]. These results might suggest that autophagosomal membranes could be involved in IBDV assembly. However, in our laboratory, we have previously studied the interplay between autophagy and IBDV infection. We determined both the level of the autophagy marker protein LC3-II in infected cells and the number of LC3-positive vesicles generated in response to viral infection, but no modifications were observed, suggesting the lack of an autophagic response after infection of cells by IBDV virions. Additionally, we evaluated the role of the Atg5 and Beclin-1 proteins, both key components of the autophagic pathway. However, no differences in the viral progeny were observed in these knocked-down cells. So, these results prompted us to conclude that under the conditions tested, the autophagy pathway did not have a fundamental role in the IBDV replication cycle (Poster in the National Virology Congress, Buenos Aires, Argentina, 2011). A feasible explanation for such discrepant observations could be due to the different viral strains. Given that autophagy is an innate immune response of the cell, it is likely that small differences in the virulence of the strain induce a substantially different response from the cell.

Of note, in the present report, we showed that IBDV infection likely causes stress to the ER, as evidenced by the concomitant induction of BiP expression and LDs accumulation. For infectious pancreatic necrosis virus, another well-characterized virus belonging to *Birnaviridae* family, it has been shown recently that the virus induces inhibition of cellular protein synthesis in permissive cells, with the involvement of phosphorylation of eukaryotic initiation factor 2 [86]. Thus, we reasoned that viral protein expression burst into the host cell may lead to the stress of the ER, with the consequent promotion of UPR and LDs accumulation. Indeed, the mechanism involved in IBDV-induced ER-stress will be further explored in our laboratory.

## 5. Conclusions

In conclusion, we showed original data demonstrating that IBDV relies on the organelles and the molecular network of the cellular secretory pathway for the establishment of replication complexes as well as the subsequent virion assembly, maturation and release. The exact nature of the membrane wrapping up the viral assembly sites remains an intriguing question in the field of IBDV, but our results point toward an ER role in this viral step. We demonstrated that newly generated viral particles organize within single-membrane compartments in close proximity to the rough ER, thereby causing ER stress.

## Data Availability

The research data is fully available upon request.
